# Unveiling the Unseen: A Rare Case of Primary Pulmonary Lymphoma

**DOI:** 10.7759/cureus.70959

**Published:** 2024-10-06

**Authors:** Maria Jose, Nalini Jayanthi, Harshavardhini P, Sowmya P

**Affiliations:** 1 Respiratory Medicine, SRM Medical College Hospital and Research Centre, Chennai, IND

**Keywords:** acanthosis nigricans, diffuse large b cell lymphoma (dlbcl), non-hodgkin's lymphoma, paraneoplastic syndrome, primary pulmonary lymphoma

## Abstract

Primary pulmonary lymphoma (PPL) is an uncommon condition involving the lungs, characterized by the abnormal clonal growth of lymphoid cells. Patients with PPL can be asymptomatic or present with vague clinical signs such as cough, fever, chest discomfort, and shortness of breath. Some may also have underlying immunosuppression or autoimmune conditions. Tuberculosis being more common with a higher incidence in endemic areas, PPL can often be misdiagnosed as tuberculosis. Also, they share common symptoms like cough, fever, fatigue, unexplained weight loss, and an upper lobe predilection. Therefore, diagnosing PPL from other common pulmonary diseases is of utmost importance in early diagnosis and treatment. Generally, small biopsy samples are essential for precise diagnosis and prompt treatment. Therapeutic options include chemotherapy, radiotherapy, immunotherapy, and surgical intervention. Herewith we outline a case initially presented as acanthosis nigricans, referred with respiratory symptoms, pleural effusion, and mediastinal lymphadenopathy, which was primarily thought to be a tuberculous pleural effusion. Apart from clinicoradiological and laboratory analysis, thoracoscopy-guided biopsy and histopathological examination pointed toward further steps in diagnosis. The patient was initiated on chemotherapy with an R-CHOP regimen. This case depicts the call for a multidisciplinary perspective for the definite and expeditious diagnosis and management of a paraneoplastic syndrome associated with diffuse large B cell lymphoma. This discussion also highlights the need for a thorough evaluation of paraneoplastic syndromes, as they are crucial in the early diagnosis of the disease and in identifying relapses.

## Introduction

Lung cancer is the most common type of cancer and the leading cause of cancer-related deaths worldwide [1]. In contrast to non-small cell lung cancer (NSCLC) and small cell lung cancer (SCLC), primary pulmonary lymphoma (PPL) is an uncommon clonal lymphoproliferative disease of the lungs [1,2]. It originates from the pulmonary parenchyma or bronchi, potentially involving hilar lymph nodes, with no evidence of extrathoracic lymphoma at initial diagnosis or within the following three months [3-5]. When the lung is the primary site of the tumor, the definition also covers multifocal mucosa-associated lymphoid tissue (MALT), non-Hodgkin lymphoma (NHL), pulmonary involvement with adjacent nodes (hilar or mediastinal), and multiorgan spread due to lymphomatoid granulomatosis, whose clonal nature remains debated [6].

PPL represents approximately 3.6% of extranodal lymphomas and less than 1% of all lung cancers [1,6]. It can be categorized into Hodgkin’s lymphoma (HL) and NHL. Within NHL, the principle subtypes include MALT, B-cell lymphoma, diffuse large B-cell lymphoma (DLBCL), and T/NK-cell lymphoma. DLBCL ranks as the second most prevalent form of PPL and the most common NHL, accounting for approximately one-third of all cases [7-10]. DLBCL might occur in any extranodal sites, the most common being gastrointestinal and bone marrow. When the lung is involved, it is termed PPL. It usually occurs in individuals in the sixth and seventh decades and often presents with non-specific symptoms, leading to a high chance of misdiagnosis, mostly in suspicion of pneumonia, tuberculosis, and lung cancer [3,7,8].

The radiologic appearance of PPL varies, showing single or multiple nodules or masses with uniform density or patchy high-density areas and lacking distinctive imaging characteristics. Currently, pathological diagnosis is primarily achieved through surgery or biopsy [11,12]. When imaging confirms that a lesion is localized and there is no distant infiltration, surgical therapy or localized radiotherapy may be viable options. Complete removal of the lesion through surgery could be beneficial for such patients. However, most PPLs exhibit lesions that are not limited to a single region and often involve lymph nodes or nearby tissues, making surgery insufficient for complete lesion removal. The R-CHOP regimen showed significant efficacy in treating infiltrative DLBCL, achieving complete remission in 70% to 80% of patients. At present, PPL is an uncommon condition with non-specific manifestations and radiological findings, which makes it difficult to diagnose, and the standard treatment protocol is still under debate. Herewith we present a case of mediastinal lymphadenopathy with pleural effusion, which was initially worked up as tuberculous pleural effusion.

## Case presentation

**Figure 1:**
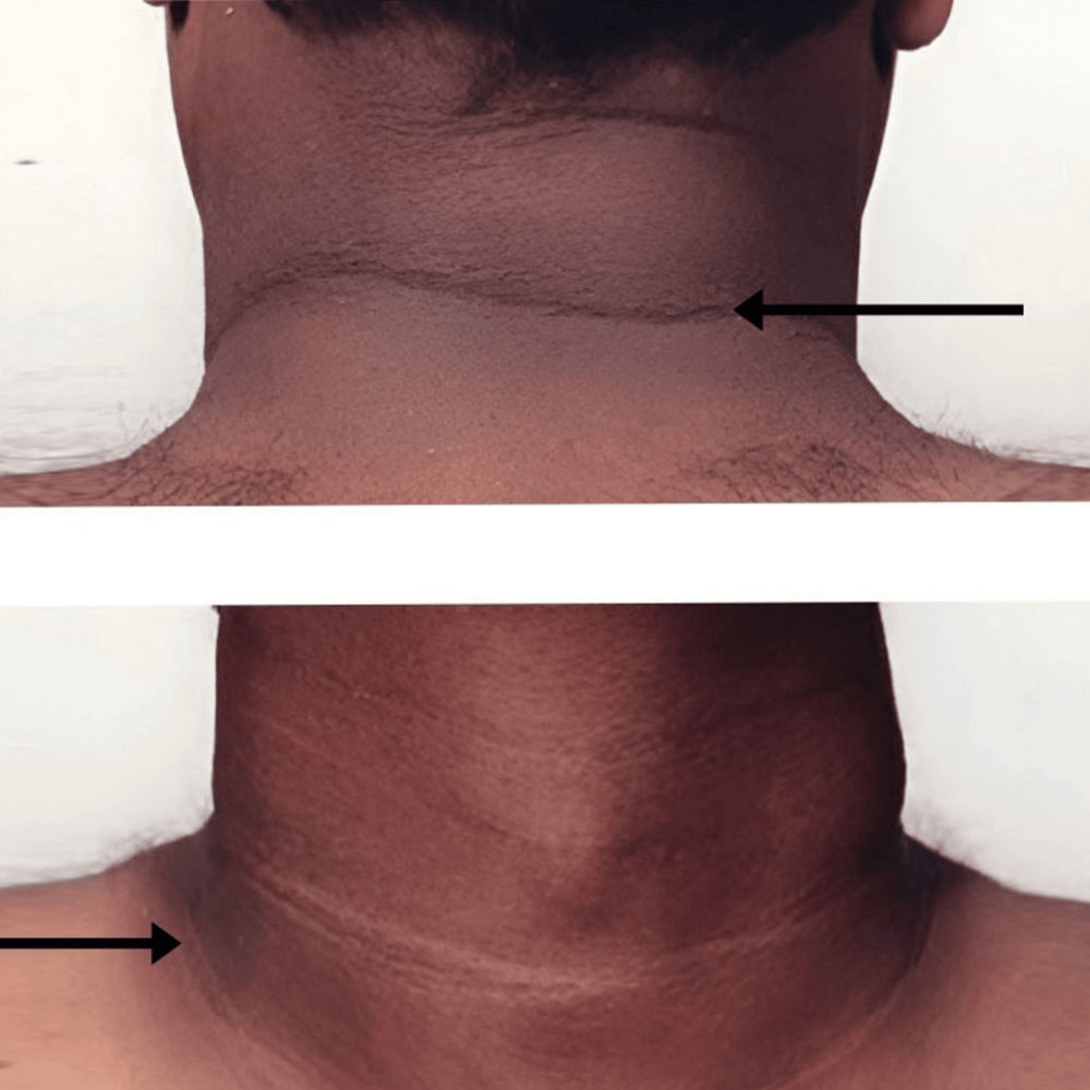
Acanthosis nigricans (AN) in the neck Velvety darkening of skin usually occurring in intertriginous areas

**Figure 2:**
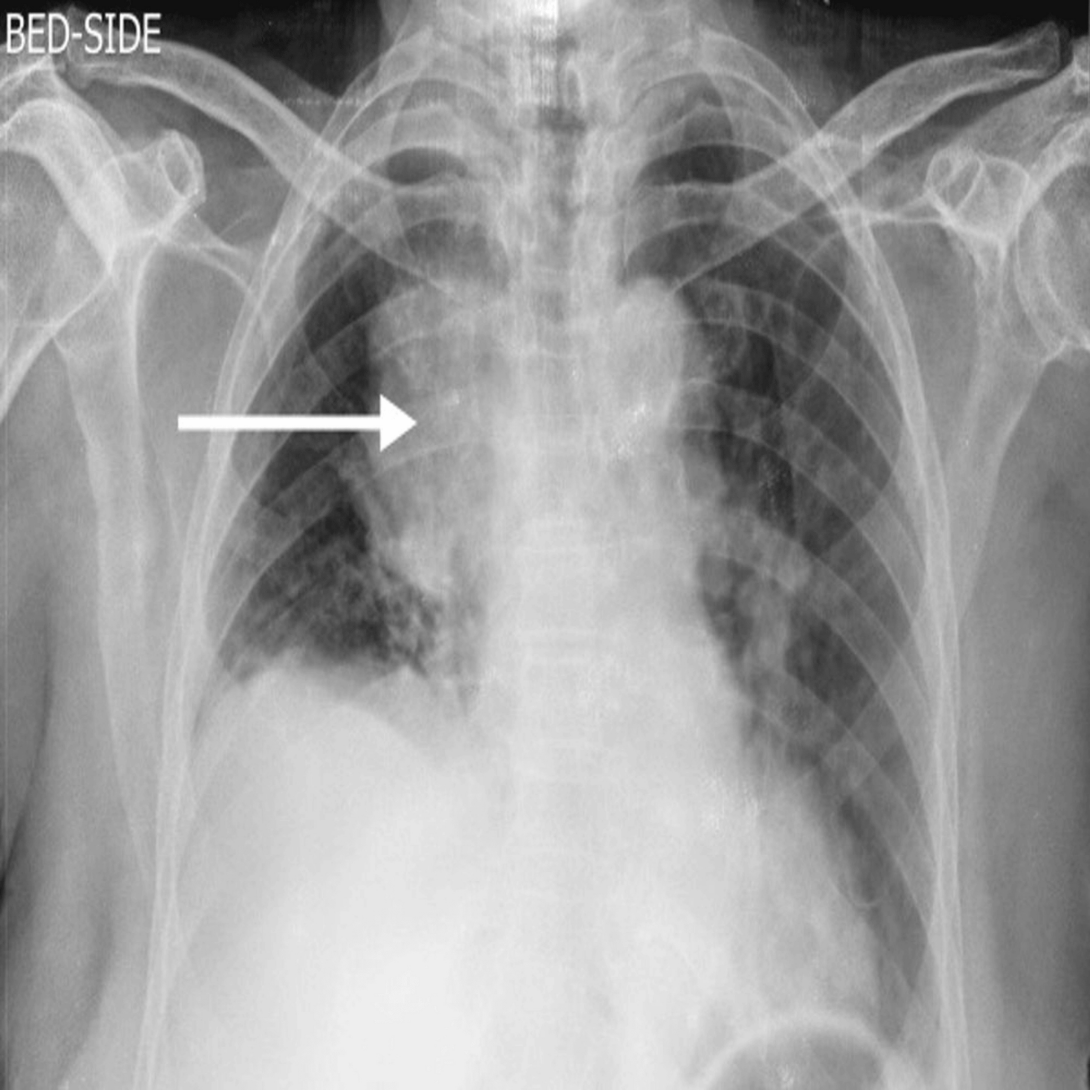
Chest X-ray showing right-sided homogeneous opacity, silhouetting right heart border

**Figure 3:**
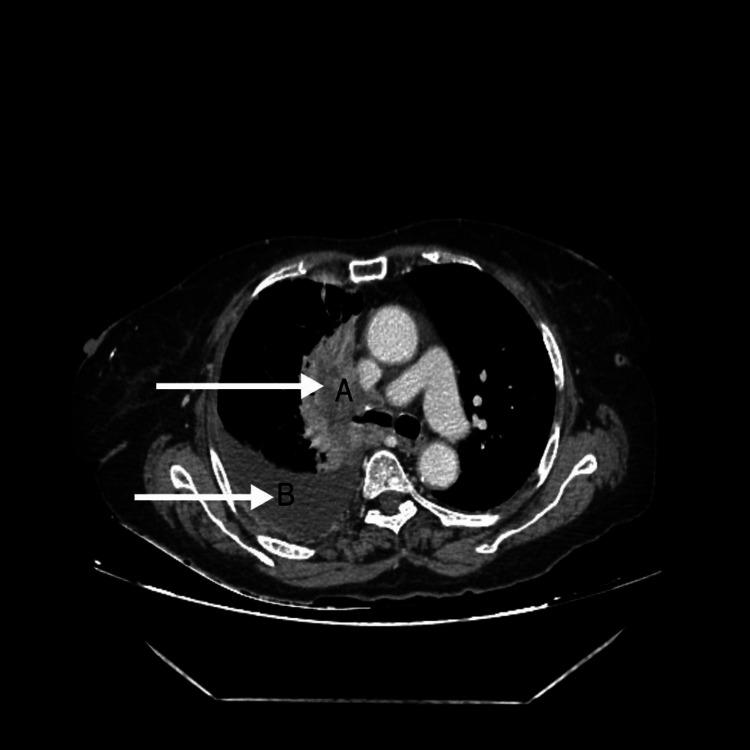
Computed tomography of chest showing right hilar mass with large discrete conglomerated lymph node measuring 71 x 39 mm in right hilar region with right pleural effusion A: Right hilar mass with enlarged hilar lymph node; B: Right-sided pleural effusion

A 55-year-old male presented to the dermatology department with complaints of darkening and thickening of skin for two months. No history of diabetes mellitus, drug intake, or familial history. He was referred to the respiratory medicine department for shortness of breath and dry cough for three days and one episode of evening rise of temperature with loss of weight. The patient was a non-smoker with no comorbidities present. He had neither been in close contact with anyone who had tuberculosis nor had any intake of anti-tuberculous drugs, according to his medical history. Furthermore, he reported that he had no previous history of allergies or any other medical history (Figures 1-3).

On physical examination, the patient presented with a pulse rate of 108 beats per minute, a respiratory rate of 22 breaths per minute, a blood pressure reading of 136/88 mm Hg, and a temperature of 98.7°F. Diminished breath sounds were noted in the lower zone of the right infra-scapular and infra-axillary areas during the chest examination. No palpable lymph nodes were detected. The abdomen was soft with no hepatosplenomegaly. Other systemic examinations were within normal limits. The patient was admitted to our department for further evaluation and management.

Chest X-ray showed right-sided homogeneous opacity, silhouetting the right heart border. Laboratory analysis indicated normal hepatic and renal parameters, along with a normal blood count. Serology results showed negative for HIV, HBsAg, and HCV. The erythrocyte sedimentation rate was 43 mm/hr. Computed tomography of the chest showed a right hilar mass with a large discrete conglomerated lymph node measuring 71 x 39 mm in the right hilar region, causing mild displacement of the right and left brachiocephalic confluence with right pleural effusion.

Thoracocentesis was done, and 1500 ml of high-colored fluid was drained. Pleural fluid analysis revealed slightly turbid yellow-colored fluid with a WBC of 1700 cells/cumm, lymphocytic predominant (95%).

Pleural fluid biochemistry showed glucose of 181 mg/dl, protein of 4 g/dl, chloride of 107 mmol/L, and lactate dehydrogenase (LDH) of 144 U/L. Pleural fluid adenosine deaminase (ADA) was 53. Based on Light’s criteria, pleural fluid was exudative (Table 1).

**Table 1:** Findings from laboratory analysis of pleural fluid LDH: lactate dehydrogenase; ADA: adenosine deaminase

Parameters	Results	References
Color	High colored	-
Appearance	Slightly turbid	-
Volume	15.0 ml	-
Reaction	Alkaline	-
WBC	1700 cells/cumm	-
Differential count	Lymphocytes - 95% Neutrophils - 5%	-
Glucose	181 mg/dl	80-140 mg/dl
Protein	4 g/dl	<3 g/dl
Albumin	2.8	0.5-1.4 g/dl
Chloride	107 mmol/L	110-125 mmol/L
LDH	144 U/L	<200 U/L
ADA	53 U/L	<40 U/L

Pleural fluid, as well as sputum acid-fast bacilli (AFB) and gene expert, were negative. Pleural fluid cytology demonstrated lymphocytic effusion with no evidence of malignant cells. Since there was no improvement and fluid was reaccumulating, the patient was planned for a biopsy.

**Figure 4:**
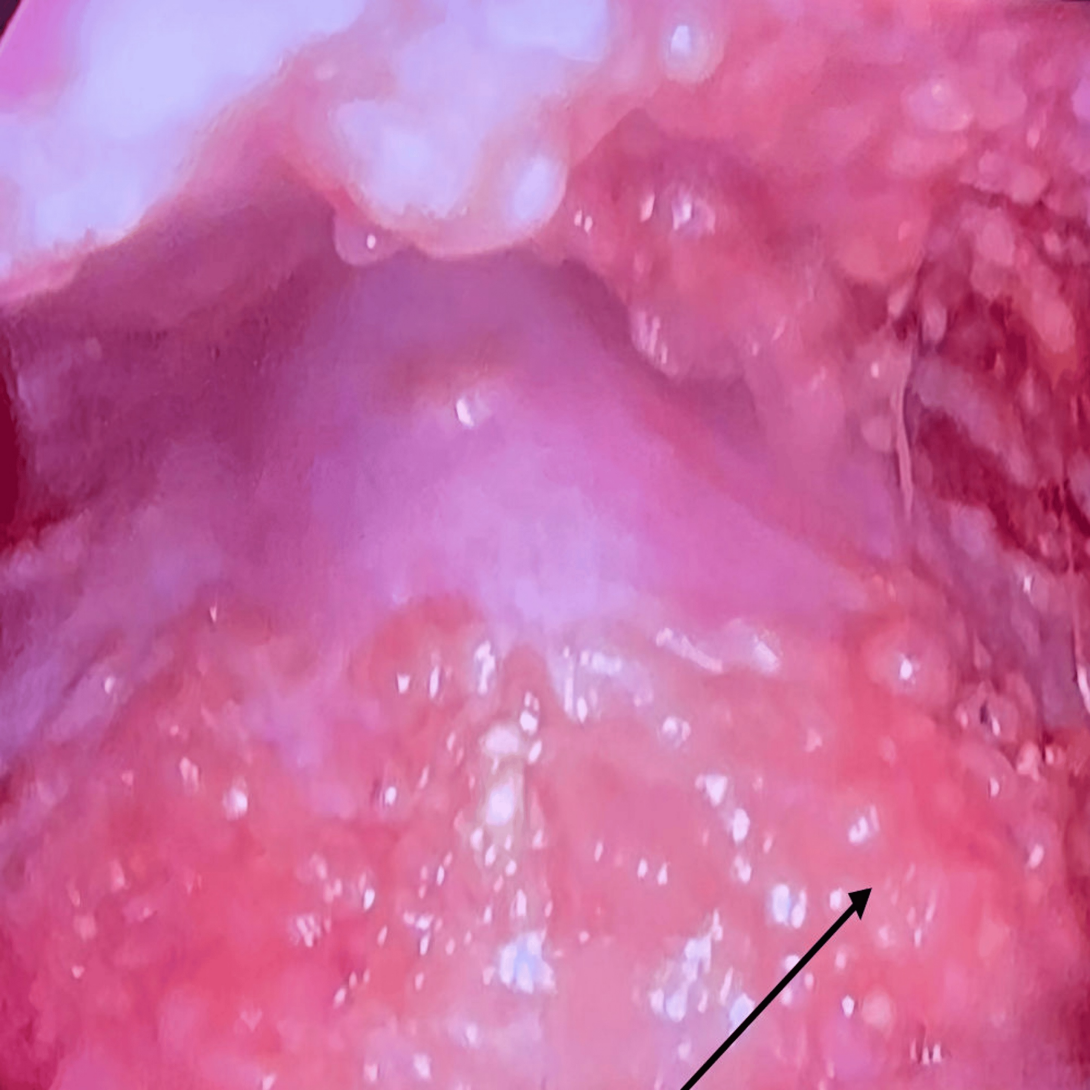
Thoracoscopy view of the patient Direct visualization of pleura revealed diffuse infiltration with multiple nodules

To confirm the diagnosis, a thoracoscopy was performed under local anesthesia with continuous cardiovascular and respiratory monitoring. The largest fluid pocket was identified using thoracic ultrasound. A horizontal incision approximately 1 cm in length was made through the skin and subcutaneous tissue at the entry site using a scalpel. Blunt dissection was then made through the chest wall into the pleural cavity. A 7-mm trocar was inserted into the pleural cavity using a corkscrew technique, followed by the removal of the obturator and the insertion of a flex-rigid thoracoscope. During the procedure, small doses of midazolam were administered intravenously. About 600 ml of pleural fluid was drained to facilitate visualization of the pleural cavity. Direct visual inspection of the pleura revealed a significant amount of bloody pleural fluid, widespread membrane hyperemia, and numerous small white nodules on the parietal pleura. Multiple biopsies were obtained from the lesions, and the samples were sent for pathological analysis. The thoracoscope and trocar were removed thereafter, and a 24F chest tube was inserted into the pleural cavity to continue draining the pleural fluid (Figure 4). There was a daily drain of about 1-1.5 l for the initial three days and then reduced to 600-650 ml daily with a total drain of 6.8 l followed by ICD removal after three weeks.

**Figure 5:**
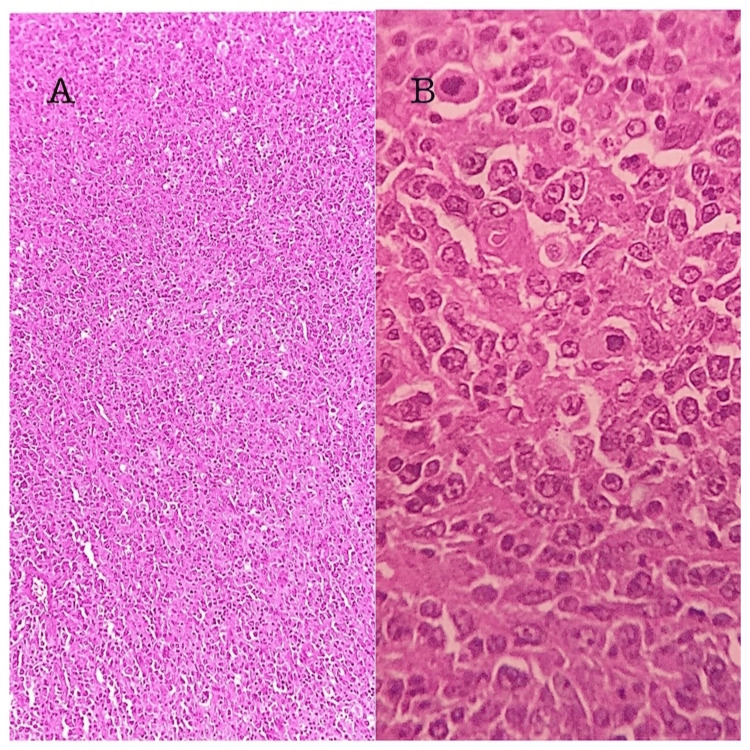
Histopathology image showing complete effacement of nodal architecture with infiltrating sheets of medium- to large-sized tumor cells A: low power view; B: high power view

Histopathological examination revealed diffuse large B cell lymphoma and was negative for MTB on gene-expert. Immunohistochemistry (IHC) markers were positive for CD20, CD3, CD15, CD45, and Ki‐67 (65-70%) (Figure 5).

**Figure 6:**
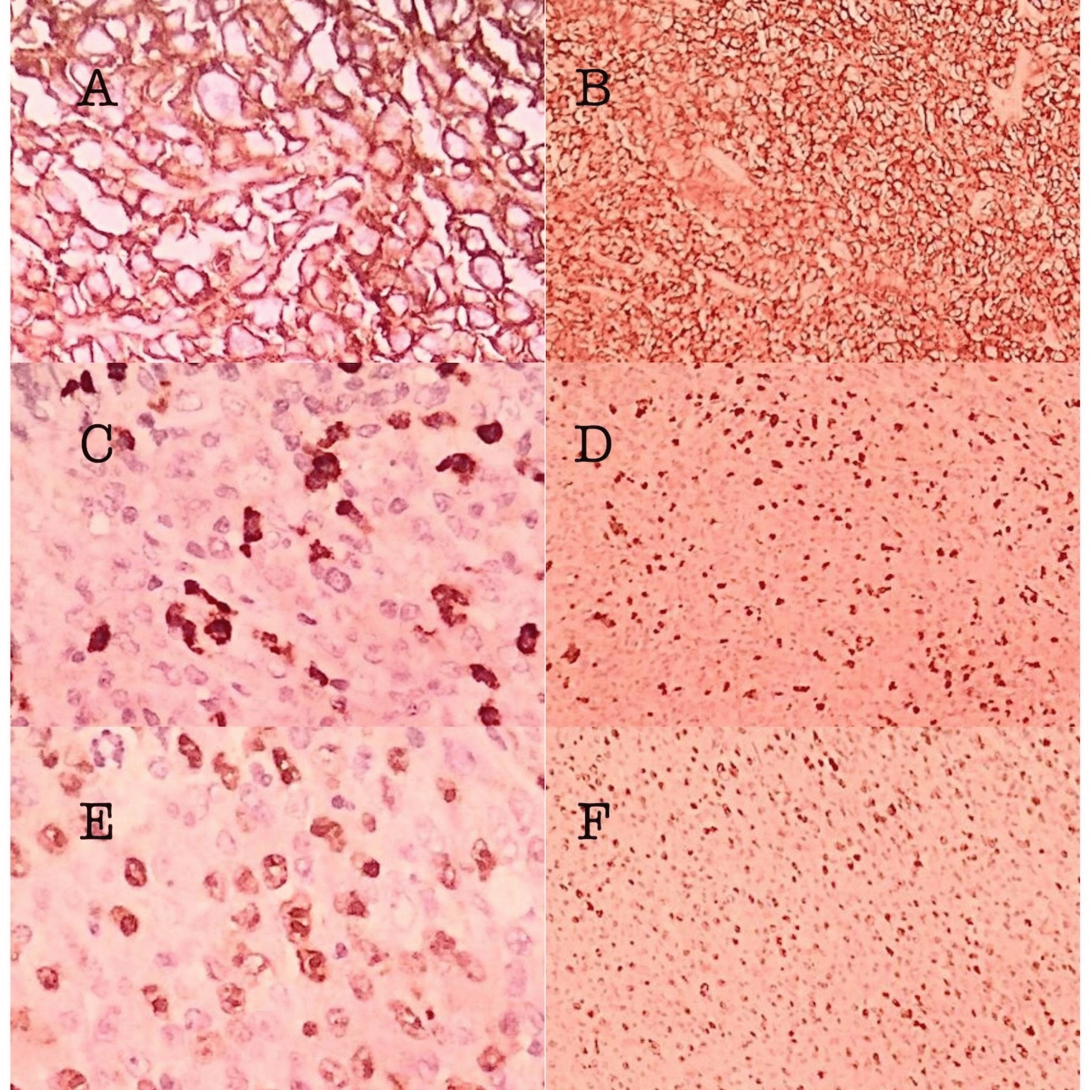
Immunohistochemistry markers A and B: CD 20 high and low power view; C and D: CD 15 high and low power view; E and F: Ki-67 high and low power view

The patient was referred to the oncology department for further management. He was given two cycles of chemotherapy (the R-CHOP regimen), after which the patient was reluctant to further treatment due to logistic reasons. The disease progressed with breathlessness and desaturation and was managed with palliative care. The patient died eight months thereafter (Figure 6).

## Discussion

Primary pulmonary diffuse large B-cell lymphoma (PP-DLBCL) is the second most prevalent type of PPL, representing 5% to 20% of all primary pulmonary B-cell lymphomas. It can arise either as a primary condition or through large cell transformation from primary pulmonary marginal zone lymphoma (PP-MZL) [8]. MALT lymphoma is the most frequently encountered type, accounting for 85-90% of PPL cases [13]. The proportion of male-to-female patients is roughly equal.

DLBCL is characterized by its aggressive nature and has various morphological forms like centroblastic, immunoblastic, and anaplastic types. These forms represent different patterns of cell appearance and behavior, which can influence diagnosis and treatment strategies. These tumors can sometimes occupy a substantial portion of a lung lobe and are frequently associated with areas of necrosis. The tumor’s growth pattern can be intraparenchymal or endobronchial and may be associated with pleural effusion and pleural masses [4,14-16]. When DLBCL primarily affects the lungs, it often presents as a solitary nodule or a sizable mass. Macroscopically, PP-DLBCL appears as a tan-white or pale yellow mass with a vaguely nodular texture and well-defined borders [3,17]. Areas of central necrosis, cavitation, and hemorrhage are commonly observed, often presenting as cavities with thick walls that can resemble abscesses [18,19].

Patients with PP-DLBCL often exhibit "B-symptoms," which include fever, night sweats, and a weight loss of at least 10% of body weight over six months. Additionally, they may experience site-specific symptoms such as chest pain, respiratory distress, shortness of breath, cough, and less commonly, hemoptysis [2,3,20]. Though initial laboratory tests might not reveal exact details about the lymphoma, a biopsy is crucial for a definitive diagnosis. The approach to the differential diagnosis of PLL is determined by the specific subtype of lymphoma. Pulmonary MALT lymphoma should be distinguished from reactive inflammatory conditions, while high-grade lymphomas can be mistaken for poorly differentiated lung carcinoma, metastatic disease, and other forms of lymphoma. Typically, small biopsy samples are adequate for accurate diagnosis and permitting timely initiation of treatment [11,21]. The diagnostic approaches include bronchoscopic biopsy, CT-guided percutaneous needle lung biopsy, video-assisted thoracic surgery (VATS), open lung biopsy, and pleural membrane biopsy. Histological samples are assessed and categorized by hematopathologists according to the latest WHO guidelines. IHC markers like CD20 are pan-B-cell markers found in most mature B-cell lymphomas, and Ki-67 indicates tumor cell proliferation by identifying cells in the cell cycle stages other than G0.

Surgical resection of PP-DLBCL is generally considered curative only for localized lesions, which are uncommon because the disease often metastasizes to the mediastinum and other extra-thoracic organs [3,8]. Chemotherapy and/or radiotherapy are typically given post-surgery because of the high likelihood of local or distant recurrence [2,18,22]. The standard treatment regimen is R-CHOP, which includes rituximab, cyclophosphamide, doxorubicin, vincristine, and steroids. Advances in treatment include the use of CAR T-cell immunotherapy, which has shown success in treating relapsed and refractory cases. Factors that are associated with a poor prognosis include age over 60, B symptoms, the presence of multiple or bilateral lung lesions, pleural effusion, and the development of cavitation. Approximately 60% of patients had a five-year survival rate without progression, and about 65% had an overall survival rate [23].

Paraneoplastic syndromes comprise a collection of signs and symptoms linked to cancer that manifest in organs and tissues distant from the primary tumor and are not caused by the direct spread of cancer cells [24]. Skin manifestations associated with internal malignancies can arise either from direct causes, such as the invasion of the skin by the primary tumor or its metastases or from indirect causes that trigger skin-related signs and symptoms as part of paraneoplastic syndromes [25]. In some instances, skin conditions may provide an early indication of an underlying internal cancer [26].

Certain dermatologic syndromes are particularly specific and can signal the presence of lung cancer. In paraneoplastic cases, therapeutic responses are generally less effective compared to their non-paraneoplastic counterparts. Acanthosis nigricans (AN) is characterized by skin thickening and hyperpigmentation, mainly occurring in the neck and axilla. In cases of paraneoplastic AN, adenocarcinoma is the most common histologic type, with 70-90% of these cancers located intra-abdominally and 55-61% being gastric adenocarcinomas [27]. Paraneoplastic AN is less commonly associated with NSCLC. The hallmark features of AN include darkened and thickened skin, particularly in the folds of the axilla, groin, and neck. The underlying cause of AN is not well understood, but one hypothesis suggests that excessive circulating insulin interacts with insulin-like growth factor receptors on keratinocytes and dermal fibroblasts, leading to the development of the condition. Moreover, increased tumor production of transforming growth factor alpha (TGF-α) may drive keratinocyte proliferation, contributing to paraneoplastic AN. While no specific treatment for AN has been established, treating the underlying malignancy often results in improvement of the skin condition [27].

## Conclusions

Diagnosing PPL is challenging without a biopsy. Histopathological examination with IHC is essential for confirmation, offering the patient a better opportunity for targeted treatment, improving prognosis, and preventing disease progression. Although rare, PPL should be considered as a differential diagnosis when assessing neoplastic pulmonary lesions.

The exact cause of AN remains unclear in this case. Hence, the role of AN as a paraneoplastic syndrome cannot be excluded. Paraneoplastic syndromes need to be carefully considered, as they play a key role in the early diagnosis of the disease and in detecting relapses. Tumor-induced secretion of growth factors, such as tumor growth factor alpha, may also lead to keratinocyte proliferation and AN development. Hence, AN cases should be thoroughly examined with skin biopsy to rule out their paraneoplastic origin. Paraneoplastic symptoms might precede the diagnosis of the malignancy or appear alongside other symptoms of the primary tumor.
